# Seroprevalence and Virus Activity of Rift Valley Fever in Cattle in Eastern Region of Democratic Republic of the Congo

**DOI:** 10.1155/2018/4956378

**Published:** 2018-06-28

**Authors:** Tshilenge M. Georges, Masumu Justin, Mbao Victor, Kayembe Jean Marie, Rweyemamu Mark, Mulumba Mfumu K. Léopold

**Affiliations:** ^1^Faculty of Veterinary Medicine, University of Kinshasa, University Street, P.O. Box 117, Kinshasa XI, Democratic Republic of the Congo; ^2^Central Veterinary Laboratory, Wangata Street, P.O. Box 8842, Gombe, Kinshasa, Democratic Republic of the Congo; ^3^Faculty of Veterinary Medicine, National Pedagogic University, Matadi/Liberation Street, P.O. Box 8815, Ngaliema, Kinshasa, Democratic Republic of the Congo; ^4^National Institute for Biomedical Research (INRB), 5345 Huileries Street, P.O. Box 1197, Gombe, Kinshasa, Democratic Republic of the Congo; ^5^International Development Research Centre, Kenya Street, P.O. Box 62084 00200, Nairobi, Kenya; ^6^Faculty of Medicine, University of Kinshasa, University Street, P.O. Box 117, Kinshasa XI, Democratic Republic of the Congo; ^7^Southern African Centre for Infectious Disease Surveillance, Sokoine University of Agriculture, Chuo Kikuu, P.O. Box 3297, Morogoro, Tanzania

## Abstract

Rift Valley fever (RVF) is a zoonotic disease that is characterized by periodic and severe outbreaks in humans and animals. Published information on the occurrence of RVF in domestic animals is very scarce in the Democratic Republic of the Congo (DRC). To assess possible circulation of Rift Valley fever virus (RVFV) in cattle in the eastern province of DRC, 450 sera collected from cattle in North Kivu, South Kivu, and Ituri provinces were analyzed using the enzyme-linked immunosorbent assay (ELISA), for the detection of viral Immunoglobulin (Ig) G and M, and reverse transcriptase polymerase chain reaction (RT-PCR), for detection of viral RVF RNA. A cumulative anti-RVF IgG prevalence of 6.22% (95% CI 4.25–8.97) was recorded from the three provinces sampled. In North Kivu and Ituri provinces the anti-RVF IgG prevalence was 12.67% [95% CI 7.80–19.07] and 6% [95% CI 2.78–11.08], respectively, while all the sera collected from South Kivu province were negative for anti-RVF IgG antibodies. Anti-RVF IgM prevalence of 1.8% was obtained among sampled animals in the three provinces. None of the positive anti-RVF IgM samples (n=8) was positive for viral RVFV RNA using RT-PCR. Our findings suggest that RVFV is widely distributed among cattle in eastern province of DRC particularly in North Kivu and Ituri provinces although the epidemiological factors supporting this virus circulation remain unknown in these areas.

## 1. Introduction

Rift Valley fever virus (RVFV) is a vector-borne single stranded zoonotic virus that infects humans and a wide range of vertebrate hosts including cattle, small ruminants, camels, and wildlife species. RVFV belongs to the family Bunyaviridae, genus* Phlebovirus *[1], and is endemic in many countries on the African continent, in the Arabian Peninsula, and some Indian Ocean Islands [2–4].

RVFV is mainly transmitted among livestock through mosquito bites although vertical transmission between animals is also reported [5, 6]. In livestock and wild animals, infections with RVFV cause significant morbidity and mortality from 10% to 30% [7]. The Rift Valley fever (RVF) infection varies greatly in severity. A typical sign of RVFV infected pregnant cows and sheep is abortion. In cattle, abortion is often the only obvious indication of infection with the virus [8]. Calves are less susceptible to the virus than lambs and the mortality rate in RVFV infected newborn lambs ranged between 95 and 100% [9]. Wildlife also plays a role in the transmission of RVFV especially for virus maintenance during enzootic periods [10–14]. Limited research has been done to detect anti-RVFV antibodies and to determine transmission patterns of diseases during interepizootic period (IEP) in wildlife mammals in Central Africa. However, published studies associated with the role of mammals orders in the epidemiology of Rift Valley fever virus were reported [15] and some other field studies were done [16–18]. RVFV antibodies have been detected among wildlife in Central African Republic, Chad, where outbreaks have not been reported [13, 19, 20]. As for animals, the virus is mostly transmitted to humans through bites of infected* Aedes* mosquitoes [21] or by contact with or inhalation of aerosols during the handling or slaughtering of infected ruminants [22]. RVFV is sometimes detected in the absence of obvious clinical manifestation like abortions during interepidemic periods (IEPs). RVF epidemics can last 5 to 15 years or between 3 and 5 years in some endemic areas [23–25].

During enzootic periods when there is normal rainfall in East Africa, it is assumed that RVFV is maintained through vertical transovarial transmission of floodwater* Aedes *species, especially in areas with shallow depression habitats or dambos [26, 27]. High viremia in infected ruminants caused by infected* Aedes *mosquitoes that emerge from flooding events may allow the spillover of RVFV to secondary vectors such as* Culex *or* Anopheles *mosquitoes [4]. Factors associated with epizootics in West Africa and high rain forest zones of coastal and Central Africa remain unknown [28, 29]. RVFV activities often occur annually and are associated with seasonal rainfall during nonepidemic periods [30]. Sporadic cases of RVFV can easily be confused with other livestock diseases which exhibit similar clinical signs. RVF is encountered in endemic and epidemic forms along the east and south coast of Africa, in West Africa, and in Madagascar. The virus has spread further north including into the Middle East [8, 31, 32].

Several sub-Saharan African tropical and subtropical countries have reported outbreaks of RVFV [33, 34]. Arboviral disease was also reported in Uganda [35, 36] and in Rwanda [37]. Furthermore, a strain of RVFV was isolated in Uganda in 1944 [38] outside of an outbreak event. In the Democratic Republic of the Congo (DRC), no outbreak has been reported [39] despite the evidence of positive RVFV serological results recorded in cattle as reported by Mulumba et al. [40]. Three provinces of eastern DRC (North Kivu, South Kivu, and Ituri) bordering Burundi, Uganda, and Rwanda constitute the Occidental Rift Valley and have been classified as survey zones for RVFV. Owners of cattle often report cases of abortion without massive deaths of young animals. The aim of the present study was to assess possible circulation of RVFV in cattle population during IEP in three high-risk provinces of eastern DRC.

## 2. Materials and Methods

### 2.1. Study Area

**Figure 1:**
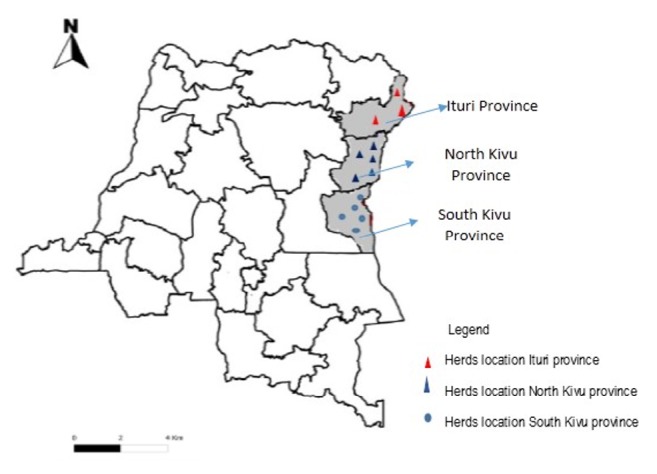


This study was conducted in three provinces (North Kivu, South Kivu, and Ituri) in unvaccinated cattle herds (Figure 1). North Kivu, South Kivu, and Ituri provinces constitute the western border of the Rift Valley and are at high RVF risk. North Kivu province is located at longitude 29°12′ to 29°17′ east and latitude 0°8′ north to 0°40′ south. The province is subdivided into 6 territories (Beni, Lubero, Butembo, Masisi, Rutshuru, Walikale) and samples were collected from five of these territories (Beni, Lubero, Butembo, Masisi, and Rutshuru). It has two different agroecological zones separated by the equator: a zone of medium altitude (1,000–1,850m above sea level, latitude 0°7′ to 0°8′ north) and a zone of high altitude (> 1,850m above sea level, latitude 0°19′ to 0°40′ south). The mean temperatures at medium and high altitudes reach 17.4 and 19.4°C, respectively, while the mean monthly rainfalls at medium altitude (256.5mm) are higher than those at high altitude (144mm).

South Kivu province is located at 3° 01′ south 28°16′ east, close to Rwanda and Burundi, and its eastern border corresponds to the western Rift Valley. The province is subdivided into 8 territories (Fizi, Idjwi, Kabare, Kalehe, Mwenga, Shabinda, Uvira, and Walungu) and samples were collected from five of these territories (Fizi, Kabare, Mwenga, Shabinda, and Uvira). The mountainous eastern part contrasts with the central and western parts of the province which have high and low plateaus, respectively. The eastern part of province (mountainous Kivu) has a mountain climate characterized by mild temperatures with a dry season lasting for 3-4 months, from June to September. The central and western parts of South Kivu province, particularly Shabunda, Mwenga, and Fizi territories, are a dense rainforest zone with an equatorial humid climate. The Ruzizi lowland is associated with a microclimate comprising a tropical climate and dry trend with low rainfall (± 1,000 mm/year). The average annual rainfall is 1,800 mm, with wide yearly fluctuations. South Kivu province has an altitude ranging from 1,800 to 3,300m. The forest is being cleared for agriculture and firewood around villages and cattle grazing on the high plateaus (e.g., Itombwe Mountains). The vegetation of this area is mostly grassland that intergrades with lowland. The average temperatures vary between 19 and 28°C.

Ituri province (one of the four districts of Orientale province in the past) is located at 01°50′ north 29°30′ east, northeast of the Ituri River and on the western side of Lake Albert. It borders Uganda and South Sudan. The province is subdivided into 5 districts (Aru, Mahagi, Djungu, Irumi, and Mambasa) and samples were collected from tree of these territories (Aru, Irumi, and Mahagi). Ituri province is on a high plateau ranged between 1,000 and 2,000m. It has a large tropical forest as well as a savanna landscape. The Ituri forest geographic boundaries are difficult to define as the forest blends in with other forests and swamp regions. The north is limited by savanna, the east by the western Rift Valley, and the south west by a lowland rain forest. The dry season lasts from December to February. The heaviest rains occur in October and early November with significant flooding. Abundant rainfall is recorded in the southwest of the province where it can reach a maximum of 2,000mm. The average temperatures range between 19 and 31°C.

#### 2.1.1. Study Design and Sample Collection

The sample size was determined according to the method described by Martin et al. [41].

*n= [1,96* ^2^ *× p (1-p)]/L* ^2^, where 1.96 is the z value for the desired confidence level (95%),* p *is an estimated expected prevalence of infection, and* L *is the tolerable error. As the prevalence of RVF in the study area is not known a priori, 50% prevalence was assumed with 5% tolerable error used. The calculated size sample for this region was 385. Extensive livestock system in this area is characterized by an average herd size of 30-50 heads of cattle. As data on cattle numbers in each district were not available at the time of sampling, three main criteria were used in selecting herds: (i) the accessibility of herds (insecure areas being excluded), (ii) a minimum herd size of 50 head of cattle of both sexes aged at least 12 months to exclude the effect of colostral antibodies, and (iii) no vaccination history.

Cattle in this region are not regularly vaccinated against RVF. With farmers' consent, 10% of cattle from each herd were randomly selected during the rainy season from November to December 2013, from which 450 blood samples were collected (150 in North Kivu, 150 in Ituri, and 150 in South Kivu). Sampled cattle were classified according to age: 1–3 years and 3–7 years. Blood samples were collected in dry vacutainer tubes from the jugular vein. Each vacutainer tube was labeled and individual information of each animal was recorded. Animals were bled from jugular vein.

The collected blood samples were kept overnight at room temperature in cool box at 4°C to allow blood clotting. On the next day, they were centrifuged at 3,000g for 10 minutes to sediment the erythrocytes. Sera were transferred into new vials and labeled before being stored at -20°C until tested.

## 3. Laboratory Sample Processing and Analysis

### 3.1. Serological Analysis Using Competitive IgG ELISA

Serological assays of all samples (n=450) were carried out using anti-RVF nucleoprotein (NP) IgG antibodies using ID screen® RVF competition multispecies ELISA (ID-Vet Innovative Diagnostics, Montpellier, France) according to the manufacturer's instructions. After adding the stop solution, the optical density (OD) was read at room temperature using an ELISA reader (Thermo Electron Corporation, Multiskan EX®, Shanghai, China) at 450nm.

For the validity of each plate, the mean value of the two negative controls (ODNC) was calculated and the plate was considered valid when ODNC > 0.7. For a valid plate, the mean value of the two positive controls divided by ODNC was < 0.3. For each sample the competition percentage (S/N%) was calculated by dividing (OD sample/ODNC) x 100. Sample was considered positive if the value was equal to or less than 40%. A value greater than 50% was a considered as negative and values between 40 and 50% indicated a doubtful result. In this study, all doubtful samples were considered negative.

### 3.2. Serological Analysis Using Capture IgM ELISA

To detect recent infection (IgM), all samples were tested using ID screen RVF IgM ELISA (ID-Vet Innovative Diagnostics, Grabels, France), as per manufacturer's instructions. The test was valid when the mean corrected value of the positive control OD (ODNC) was greater than 0.35 and the ratio of the mean corrected values of the positive and negative controls (ODNC and ODPC) was greater than 3. The sample was considered positive when the percentage of positivity was greater than or equal to 50%, doubtful when between 40% and 50%, and negative when less than or equal to 40%. All doubtful samples were considered as negative in this study.

### 3.3. Molecular Test

The QIAamp UltraSens Virus Kit provided by QIAGEN® is designed for rapid, highly sensitive, and efficient recovery of viral RNA and DNA from plasma or serum. QIAamp UltraSens was used for the detection of viral nucleic acids in positive IgM serum following the manufacturer's instructions. The SuperScript™ One-Step reverse transcriptase polymerase chain reaction (RT-PCR) with Platinum®* Taq* provided by Invitrogen life technologies (Invitrogen, USA) was used to run RT-PCR. The sequences of the set of primers used were NSa: CCTTAACCTCTAATCAAC and NS2g: TGATTTGCAGAGTGGTCGTC.

PCR amplification was completed using an Gen Amp® machine (PCR System 9700, Singapore) as follows: 50°C for 30 minutes: 1 cycle; 95°C for 10 minutes: 1 cycle; 95°C for 15 seconds, 50°C for 30 seconds, 72°C for 45 seconds: 40 cycles; 72°C for 5 minutes: 1 cycle. 10*μ*l of the amplified product was visualized on 1% of agarose gel with ethidium bromide staining under UV light.

### 3.4. Statistical Analysis

Data from the competition and Capture ELISA were analyzed using Statistical Package for Social Sciences (SPSS) version 15.0 and Excel (MS Excel 2007) to calculate the percentage of RVF antibodies. Herds, age, and territory were used as explanatory factors. Confidence intervals of the proportions of detected antibodies were calculated at 95% confidence and a 5% level of significance was used. Chi-square (*χ*2) was used to detect any association between antibody detection and herds, ages (1–3 and >3–7 years), and location.

## 4. Results

A total of 450 sera samples were analyzed, among which 28 were positive for RVFV-IgG antibodies. This finding represents an anti-RVF IgG antibodies prevalence of 6.22% (95% CI 4.25-8.97) as shown in Table 1. An anti-RVF IgG antibodies prevalence of 12.67% (95% CI 7.80-19.07) and 6% (95% CI 2.78-11.08) were calculated for North Kivu and Ituri provinces, respectively. None of the sera collected in South Kivu was positive for anti-RVF IgG antibodies. There was a significant difference (*p*<0.05) in prevalence between North Kivu and Ituri provinces. Within North Kivu province, there was no statistically significant difference in the prevalence (*p*=0.81, *χ*^2^:1.61) among territories. However, statistical differences were observed (*χ*^2^:13.47,* p*<0.0012) among the territories of Ituri province. There was no significant difference in prevalence between male and female cattle (RR: 0.2491 [0.0356-1.7446], *χ*^2^: 2.5468,* p*=0.111) as shown in Tables 2 and 3.

All samples collected were screened for IgM ELISA. The results showed that 1.8% (8/450) of cattle had anti-RVF IgM antibodies. In North and South Kivu provinces 2.0% (3/150) of cattle had anti-IgM antibodies while, respectively, compared to 1.3% (2/150) that was obtained in Ituri province. No statistical difference (*χ*^2^: 0.254,* p*>0.880) was observed in those three provinces. None of the positive anti-RVF IgM samples (n= 8) was positive for viral RVFV RNA using RT-PCR.

## 5. Discussion

This study was designed to investigate the circulation of RVF in the Occidental border of Rift Valley. In DRC this area comprises three provinces: Ituri, North Kivu, and South Kivu. In total 450 head of cattle were screened for the presence of anti-RVF IgG antibodies and anti-RVF IgM antibodies, and positive anti-RVF IgM samples were analyzed for the presence of RVFV RNA using RT-PCR technique. Anti-RVF IgG ELISA technique was used to assess the level of exposition of cattle to RVFV in the region since antibodies last for a long period of time while anti-RVF IgM and RT-PCR techniques served for the detection of recent infections.

Results of this study revealed that 6.22% of cattle were positive for anti-RVF IgG in the three provinces. In North Kivu, the prevalence was 12.66% while 6.0% of cattle had anti-RVF IgG in Ituri province. None of the cattle sampled in South Kivu were found with anti-RVF IgG. Higher proportions of anti-IgG-positive sera were observed in Masisi (North Kivu) and Aru (Ituri) territories. Apart from villages, no antibody was found; the prevalence in the rest of the villages ranged from 2% to 16%. The presence of IgG-positive cattle in most of these villages is an indication of a previous contact between these animals and RVFV. Since young animals aged less than three years old were also found and in a larger proportion than adults, with IgG antibodies it can be assumed that the contact with the virus occurred in situ. This can be supported by the circulation of anti-RVF IgM antibodies in the area whereby 1.8% of cattle were found with such antibodies in the three provinces.

In these areas no history of RVF outbreak has so far been documented, so the presence of RVF IgM antibodies in the absence of clinical signs may indicate silent circulation of the virus in the region. Indeed silent circulation of RVFV has previously been described elsewhere in sub-Saharan Africa and suggests that, in some locations, the virus may circulate for decades in the absence of reported outbreaks or identification of clinical cases in humans or animals. In previous reports, virus circulation occurring among apparently healthy humans and animals has been noticed in Somalia [42], Uganda [43], Mozambique [44], Kenya [45], Tanzania [24, 45], Mauritania, Senegal [46, 47], and elsewhere [48–50]. Similarly RVF antibodies have also been detected in Kenya and Mozambique, respectively, in areas where the disease has never been reported [51, 52].

The results obtained in this study showed that North Kivu and South Kivu had anti-RVF IgM antibodies prevalence of 2.0%, respectively, while Ituri province had 1.3% of prevalence. Anti-RVF IgM antibodies were detected in Butembo and Rutshuru in North Kivu, Aru, and Mahagi in Ituri province where some animals were IgG-positive. However, anti-RVF IgM antibodies positive cattle were also found in Shabunda and Kabare in South Kivu but in absence of anti-RVF IgG antibodies. Since IgG antibodies last longer than IgM antibodies [53], the presence of IgG antibodies in the absence of IgM antibodies indicates previous contact with the virus in an area where viruses no longer circulate in animals. However, if IgM antibodies are found in animals in the absence of IgG antibodies, this may indicate a recent natural exposure to the virus in the region. The presence of anti-RVFV IgM antibodies in sera tested in the present study can thus be interpreted as a possible recent infection of these animals despite the fact that none of these IgM-positive samples were found with viral acid nucleic RVF using RT-PCR. The absence of viral nucleic acid in positive IgM antibody animals can be explained by the very short lifespan of viruses in resistant animals following infection and self-cure while IgM antibodies can last longer following the removal of viruses in the body. Indeed, previous studies have shown that anti-RVFV IgM antibodies were lost in 50% of animals by 45 days after infection and were absent after two or three [1] or four [54, 55] months after infection. Our results indicate that viraemic activity in this study area was very limited during the sampling moment. Once more, these results support the existence of possible silent circulation of RVFV in cattle although the mechanism of silent circulation of RVFV in various areas is still not well known. A longitudinal cohort study involving a large number of animals is needed to clarify the epidemiology of this disease in the current study area.

Whether the distribution of RVF differs according to age and gender was also assessed in the current study. In previous reports, a correlation between seropositivity and age has been noted in livestock [56] and humans [31]. However, studies conducted by Jeanmaire et al. [57] and Sumaye et al. [24] reported that the animal's age is naturally the indicator of RVF endemicity in the area since young animals are less exposed than older animals. Indeed data from the current study revealed a significant difference in seropositivity between young animals (1–3 years old) and adults (>3–7 years old). However, results from our study showed that young animals were more infected than adults suggesting that if high prevalence is found in adults compared to their counterpart young animals in endemic areas [24, 57], or even nonendemic areas, young animals may be as infected as adults or even more as shown in this study. However since very few animals were used in our study, there is a need for conducting such survey using a large number of animals to confirm this observation. On the other hand, in agreement with the study conducted by Sumaye et al. [24] and Ahmed et al. [58], no significant difference was associated with the seropositivity between male and female in all provinces although, in our study, the lack of a significant difference may be attributed to small sample sizes. Further studies are required.

To the best of our knowledge, no RVF vaccination campaign has so far been conducted in the provinces where these animals were sampled. As a result all IgG-positive animals were originated from unvaccinated herds suggesting the occurrence of natural exposure. The distribution of RVFV in the study area highlighted spatial variations whereby higher prevalence was found in North Kivu compared to the Ituri and South Kivu provinces. Varying prevalence of RVF was previously reported in other African countries such as 9.35% recorded in Cameroon [46], 25.8% in Madagascar [57, 59], 36.6% in Mozambique [52], and 16.8% in Rwanda [60]. Similarly when our results were compared with previous works in the region, a higher prevalence of IgG antibodies (12.6%) was recorded in North Kivu compared to the 4.0% prevalence reported previously by Mulumba et al. [40] in the same province. Coupled with the spatial distribution, this time variation suggests the persistence, increase, and even spread of RVF in the region. Indeed, the increased number of cattle population from 2011 in North Kivu and Ituri provinces indicates the revival of livestock trading activities from the neighboring countries Kenya, Rwanda, Uganda, and Tanzania after instability in this part of country. However, livestock trading practices expose the DRC to virus introduction through imported cattle such as those originating from Kenya, Tanzania, and where RVFV is known to be endemic in some regions [24, 51, 61]. Since the virological or immunological status of these animals is even not checked at the border when entering the country, such practices will even more contribute to the spread of RVF in various provinces.

In conclusion, this preliminary study illustrates the increase circulation and spread of RVFV in eastern provinces of DRC. For a better understanding of RVF epidemiology in this part of the country, more studies need to be conducted including monitoring and investigation of the vector dynamics as well as livestock movements within eastern DRC and across the borders shared with RVF endemic countries.

## Figures and Tables

**Table 1:** Anti-RVF IgG and IgM prevalence in territories of Eastern regions in the DRC.

Provinces	Territories	Total tested	IgG		IgM
			Prevalence (n)	[95% CI]	Prevalence (n)
	Beni	35	14.28 (5)	(4.81-30.26)	0.0 (0)
	Lubero	41	12.28 (5)	(4.08-26.20)	0.0 (0)
North Kivu	Butembo	21	4.71(1)	(0.12-23.82)	4.8 (1)
	Masisi	38	15.79 (6)	(6.02-31.25)	0.0 (0)
	Rutshuru	15	13.3 (2)	(1.66-40.46)	13.3 (2)

Sub-total		150	12.67 (19)	(7.8-19.07)	2 (3)

	Aru	50	16.0 (8)	(7.17-29.11)	2.0 (1)
Ituri	Irumi	50	0.0 (0)	(0.0-7.11)	0.0 (0)
	Mahagi	50	2.0 (1)	(0.05-10.65)	2.0 (1)

Sub-total		150	6 (9)	(2.78-11.08)	1.3 (2)

	Fizi	13	0.0 (0)	(0.0-24.71)	0.0 (0)
	Kabare	37	0.0 (0)	(0.0-9.49)	2.7 (1)
South-Kivu	Mwenga	39	0.0 (0)	(0.0-9.03)	2.6 (1)
	Shabunda	28	0.0 (0)	(0.0-12.34)	7.1 (1)
	Uvira	33	0.0 (0)	(0.0-10.58)	0.0 (0)

Sub-total		150	0	-	2 (3)

TOTAL		450	6.2 (28)	(4.25-8.97)	1.8 (8)

**Table 2:** Anti-RVF IgG seroprevalence according to group ages of cattle in the Eastern region in DRC using IgG ELISA test.

Age	Total tested	Total Positive	Prevalence (%)	*X* ^2^	*p*
1-3 years	226	16	7.08	0.572	0.0449
>3-7 years	224	12	5.36		

**Table 3:** Anti-RVF IgG seroprevalence according to gender of cattle in the Eastern region in DRC using IgG ELISA test.

Age	Total tested	Total Positive	Prevalence (%)	*X* ^2^	*p*
Male	60	1	1.67	1.6438	0.089
Female	390	27	6.92		

## Data Availability

The data used to support the findings of this study are available from the corresponding author upon request.
